# *Wza* gene knockout decreases *Acinetobacter baumannii* virulence and affects Wzy-dependent capsular polysaccharide synthesis

**DOI:** 10.1080/21505594.2019.1700659

**Published:** 2019-12-27

**Authors:** Tianshui Niu, Lihua Guo, Qixia Luo, Kai Zhou, Wei Yu, Yunbo Chen, Chen Huang, Yonghong Xiao

**Affiliations:** a Collaborative Initiative Center for Diagnosis and Treatment of Infectious Diseases^,^ State Key Laboratory for Diagnosis & Treatment of Infectious Diseases^,^ the First Affiliated Hospital, college of Medicine, Zhejiang University, Hangzhou, China; b Hangzhou Red Cross Hospital/Zhe Jiang Chinese Medcine and Western Medcine Integrated Hospital, Hangzhou, China; c Zhejiang Provincial People’s Hospital, People’s Hospital of Hangzhou Medical College, Hangzhou, China

**Keywords:** *Acinetobacter baumannii*, virulence, *wza*, gene knockout, gene complementation

## Abstract

To investigate the virulence of capsular polysaccharide export protein (Wza) in carbapenem-resistant *Acinetobacter baumannii* and its effect on capsule formation.

*wza* gene knockout and complementation strains were constructed, and changes in bacterial virulence were observed using *in vitro* adhesion, antiserum complement killing, anti-oxidation experiments, and infections in *Galleria mellonella* and mice. The effect of *wza* knockout on the genes *wzb* and *wzc* and *wzi* were assessed by RT-PCR.

We successfully constructed *wza* knockout and complementation strains. Compared with wild-type (WT) strains, *wza* knockout strains displayed lower adhesion to A549 cells (*p* = 0.044), lower antiserum complement killing ability (*p* = 0.001), and lower mortality of *G. mellonella* (*p* = 0.010) and mice (*p* = 0.033). Expression levels of *wzb, wzc* and *wzi* were decreased in *wza* knockout strains. The antioxidant capacity of Wza knockout bacteria was only slightly decreased. Complementation of the *wza* gene returned the adhesion ability, antiserum complement killing ability, and mortality of *G. mellonella* and mice to WT levels. Expression of *wzb, wzc* and *wzi* was also returned to WT levels following *wza* complementation.

The results clearly demonstrate that Wza is toxic. Wza affects the expression of other proteins of the Wzy capsule polysaccharide synthesis pathway, which affects the assembly, export, and extracellular fixation of capsular polysaccharide, resulting in synergistic effects that decrease bacterial virulence.

## Introduction

*Acinetobacter baumannii* (AB) is a non-fermentative, oxidase-negative, non-flagellate Gram-negative bacterium that is widely distributed in nature and considered a conditional pathogen [1]. It can cause a variety of clinical infections including pneumonia, meningitis, bacteremia, skin and soft tissue infections, peritonitis, and catheter-related and urinary tract infections [2]. Due to increases in the number of invasive surgical operations, and the wide use of antibiotics and hormones, multi-drug-resistant AB (MDRAB) is increasing in prevalence, as is the mortality rate of patients. Worryingly, there are few novel antibacterial drugs for treating these so-called superbugs, hence understanding the mechanisms of virulence of MDRAB could be important for developing effective new antibacterial drugs.

In recent years, reports on highly virulent AB have increased. Jacobs et al. (2014) reported the highly virulent AB5075 strain. The survival rate of *Galleria mellonella* and mice following AB5075 infection is consistently lower than 25%, and much lower than with other infectious strains [3]. Harris et al. (2013) also reported the highly virulent LAC-4 strain, and infection with this strain resulted in all C57BL/6 and BAMH/C mice dying within 48 h at a dose of 10^8^ colony-forming units (CFU) [4]. Jones et al (2015) also reported some highly virulent strains that were 10 to 100 times more concentrated in the lungs of infected mice than normal strains [5]. In China, there have also been reports of highly virulent strains. Liu et al. (2016) isolated the highly virulent AB strain CCGGD201101 from diseased chickens, and virulence experiments using healthy chicks confirmed that it was more toxic than AB ATCC17978 [6].

Studies on the virulence of AB have identified various virulence factors including outer membrane protein A (OmpA) [7,8], phospholipase D [9], capsular polysaccharide [10], biofilm formation-related protein (Bap) [11], O-glycosylation system protein [12], Acinetobacter trimeric transporter (Ata) [13], the Csu chaperone usher-type pilus [14], the iron acquisition system [15], and secretion of serine proteases [16]. The main virulence mechanisms include iron uptake systems, biofilm formation-related proteins, and damage repair systems [17]. Although these findings have expanded our understanding of the virulence of AB, it remains necessary to identify key virulence factors that promote bacterial homeostasis and cause disease.

The capsular polysaccharide on the surface of AB is considered an important virulence factor because bacteria evade or counteract host immune responses through capsular polysaccharides. Capsular polysaccharides play a key role in biofilm formation and surface colonization [18]. The synthesis of capsular polysaccharide is dependent on the Wzy capsule polysaccharide synthesis pathway [19,20]. Wza is an outer membrane protein encoded by the *wza* gene. Wza is responsible for transporting capsular polysaccharides from the periplasmic space to the surface of the bacterium, and it participates in the formation of bacterial capsules [21]. Homologues of Wza are found widely in Gram-negative bacteria, and Wza is reported to be a toxic factor in *Escherichia coli* [22], *Klebsiella pneumoniae* [23], *Riemerella pestis* [24], and *Vibrio alginolyticus* [25]. Other studies have reported the properties of Wza molecules in AB. Russo et al. constructed the *wza* mutant with Tn mutagenesis and its complementary derivative, and showed that *wza* mutants were capsule deficient by Western analysis. The *wza* mutants showed significantly decreased survival in human ascites fluid, human serum and in a rat soft tissue model compared to that of WT strains [18]. Similarly, Skerniškytė J et al constructed a *wza* mutant and showed that the *wza* mutant displayed an increase in serum-sensitivity and an impairment in CPS synthesis compared with the parent strain [26]. A study from our group reported finding the *wza* gene in the highly virulent strain Lac-4 and other highly virulent strains, and that the *wza* gene is an important toxic factor of AB [27]. To understand further the role of Wza in capsule formation in highly virulent AB strains from clinical isolates, we constructed *wza* knockout and complementation strains by homologous recombination and gene complementation, respectively, and assessed changes in capsular growth, biofilm formation, and infection *in vivo* and *in vitro*. We also measured the expression levels of the RNA *wzb, wzc* and *wzi*. The results provide a basis for understanding the role of Wza in the virulence of AB.

## Materials and methods

### Strains, plasmids, reagents, and culture media

Bacterial strains and plasmids used in this study are listed in Table 1. The control strain AB ATCC17978 was purchased from the American Type Culture Collection, USA. The virulent AB strain SKLX024256 was isolated from the First Affiliated Hospital of Zhejiang University School of Medicine, and the multilocus sequence type (Oxford MLST scheme) was 195. Bacterial culturing was performed using Mueller-Hinton Agar (MHA) medium (Cat. No. CM0337B; OXOID) or Mueller-Hinton Broth (MHB) medium (Cat. No. CM0405; OXOID). A bacterial genome extraction kit (Cat. No. 51,306; QIAGEN), a plasmid DNA extraction kit (Cat. No. 12,143; QIAGEN), a gel extraction DNA purification kit (Cat. No. 9762; TaKaRa), a reverse transcription kit (Code No. RR037A; TaKaRa), and an RT-PCR SYBR Green Supermix kit (Cat. No. 170–8894; Bio-Rad) were employed. PCR amplification was achieved using DNA polymerase (TaKaRa, Code. No. R045A). The amplified gene fragment was ligated with the vector using recombinant enzyme Exnase II (ClonExpress II, C112-01/02; Vazyme). Bacterial RNA was extracted using a PureLink RNA Mini Kit (Cat. No. 12183018A; Invitrogen). All strains and plasmids are listed in Table 1. All primers were designed and synthesized by Biosune company, and are listed in Supplementary Table S1 and Table S2.

**Table 1.** List of bacterial strains and plasmids used in this study.

Strain or plasmid	Relevant characteristics	Reference or source
*Acinetobacter baumannii* strains		
SKLX024256 (WT)	Wild-type clinical MDR isolate	Lab stock
WT-*Δwza*	WT with deletion in *wza* operon	This study
WT-C-*Δwza*	WT with deletion in *wza* operon and carrying PBAD33-TC^R^-*wza*	This study
ATCC 17978	Reference strain	ATCC
*Escherichia coli* strains		
DH5α	F-Φ80lacZΔM15Δ(lacZYA-argF) U169 recA1 endA1 hsdR17 phoA supE44λ–thi-1 gyrA96 relA1	Invitrogen
β2163	(F−) RP4-2-Tc::Mu dapA::(erm-pir) [KmR EmR]	[56]
Plasmids		
pLP12	oriT_RP4_ oriV_R6K_ vmi480 P_BAD_ (tetracycline resistance)	[57]
pLP12-*wza*(Up/Down)	pLP12 containing a 622bp UP *wza* upstream fragment and 647bp DOWN *wza* downstream fragment	this study
PBAD33-TC^R^	pBAD33 vector with tetracycline resistance	[58]
pBAD33-TC^R^-*wza*	PBAD33-TC^R^ carrying *wza*	This study

### Wza *gene knockout*

Gene knockout was performed as previously described [28,29]. Primers *wza*-MF1/*wza*-MR1 (Table S1) were used to amplify the homologous arm A fragment upstream of the AB SKLX024256 *wza* gene. Primers *wza*-MF2/*wza*-MR2 (Table S1) were used to obtain the homologous arm B fragment downstream of the AB SKLX024256 *wza* gene. The A and B fragments were used as templates to carry out overlapping PCR amplification. The AB fragment was purified and ligated with the suicide vector pLP12 using the recombinant enzyme Exnase II. The resulting recombinant plasmid was transformed into competent *E. coli* DH5α cells and transformed cells were spread on MH plates (12 μg/mL TC, 0.3% D-Glucose) . Positive recombinant clones containing the AB fragment were screened using primers pLP-UF/pLP-UR. After screening, the positive recombinant clone was cultured, and plasmid pLP12-*wza* was extracted and purified, and transformed into competent *E. coli* β 2163 cells. Cells were spread on an MH plate (12 μg/mL TC, 0.3 mM DAP, and 0.3% D-Glucose), and positive clones were picked.

*E. coli* β 2163 cells carrying pLP12-*wza* and AB SKLX024256 cells were cultured overnight. Then, 100 μL culture was centrifuged and the supernatant was removed. The cell pellet was resuspended in 10 μL MH medium, spread on an MH plate (0.3 mM DAP + 0.3% D-Glucose), and incubated at 37°C for 6 h; and then the cells were resuspended in 1 mL MH medium. A 100 μL sample was then spread on an MH plate (12 μg/mL TC + 0.3% D-Glucose). Only AB cells containing the insertion in the designated site of the chromosome were able to survive. Clones were picked and streaked on MH plates (12 μg/mL TC + 0.3% D-Glucose) to obtain a pure clone. The pure clone was cultured and the insertion mutation was detected using primers *wza*-TF/*wza*-TR.

One insertion mutant was spread on an MH plate (0.3% D-Glucose) and incubated overnight, and then one clone was streaked on an MH plate (0.4% L-arabinose), incubated overnight, and checked using primers *wza*-TF/*wza*-TR, and the PCR product was submitted for sequencing.

### Wza *gene complementation*

Gene complementation was performed as previously described [30,31]. The *wza* gene fragment was amplified using primers *wza*-RF/*wza*-RR. Using the pBAD33-TC^R^ plasmid as template, primers pBAD33-TC^R^-ZF/pBAD33-TC^R^-ZR were used to amplify the pBAD33-TC^R^ vector fragment, and the product was ligated with the fragment amplified from the pBAD33-TC^R^ vector using ligation recombinase Exnase II . The recombinant plasmid was transformed into competent *E. coil* DH-5α cells, and transformed cells were spread on an MH plate (12 μg/mL TC) and cultured. After screening of recombinant clones, the pBAD33-TC^R^-*wza* vector was successfully obtained. The pBAD33-TC^R^-wza plasmid was used to transform competent cells of *E. coli* β 2163 cells, and transformed cells were spread on an MH plate (TC = 12 μg/mL, DAP = 0.3 mM) and incubated overnight. Positive clones were screened and identified as *E. coli* β 2163-[pBAD33TC^R^-*wza*]. AB *wza* knockout strains and *E. coli* β 2163 -[pBAD33TC^R^ -*wza*] were cultured overnight, and 100 μL culture was centrifuged, resuspended in 30 μL MH medium, spread on an MH plate (0.3 mM DAP), and incubated at 37°C for 6 h. Cells were resuspended in 1 mL MH medium, spread on an MH plate (12 μg/mL TC), and incubated overnight; the recombinant clone was screened, and the PCR product was submitted for sequencing.

### Growth assay

The growth assay was performed as previously described [6]. AB strains cultured overnight in MH broth were normalized to an OD_600_ of 0.02 in the same medium and grown at 37°C for 18 h with vigorous aeration (220 rpm). The culture cell density was determined every hour by measuring OD_600_. The growth rates were estimated using Graphpad software.

### Transmission electron microscopy (TEM)

After centrifugation of a 3 mL overnight culture was centrifuged. The supernatants were again discarded, and the pellet was resuspended in PBS (0.1 mol/L, pH 7.4) containing 2.5% glutaraldehyde and incubated at 4°C overnight. After three washes with 0.1 M PBS and fixing with 1% citrate fixative for 1 h, samples were rinsed three times with water and dehydrated using a gradient of alcohol (50%, 70%, 90%, and then 100%). Embedding agent and pure acetone (1:1, 3:1) were added for gradient embedding, and samples were finally mixed with pure embedding agent and incubated at 37°C for 12 h, 45°C for 12 h, and 60°C for 48 h. Samples were sliced using an ultra-thin trimming machine and observed under a Philips TECNAI-10 operated at 80 kV.

### Bacterial biofilm test

Bacteria were cultured in LB medium for 14 h and diluted with MH medium to an absorbance at 600 nm (OD_600_) of 0.5, and 1 mL was placed on a NEST glass plate, incubated for 48 h at 37°C, and rinsed three times with PBS. A 6 μM sample of FITC-conA dye (Invitrogen, L13152) was then added and incubated for 30 min, and samples were rinsed three more times with PBS and observed under a confocal microscope. AB ATCC17978 served as the control strain.

### Antioxidant test

An overnight bacterial culture was diluted to OD_600_ = 0.5 and spread evenly on an MH plate, and a 6 mm circular piece of filter paper was placed on top. A 20% H_2_O_2_ solution was then dripped on to the filter paper and plates were incubated at 37°C overnight. The inhibition zone was measured using Vernier calipers. AB ATCC17978 served as the control strain.

### Anti-complement killing test

Serum was collected from healthy mice and centrifuged to obtain normal mouse serum. This was placed in a water bath at 56°C for 30 min to inactivate complement, generating inactive serum. An overnight bacterial culture was diluted to a cell density of 2 × 10^6^ CFU/mL, and normal and inactivated sera (180 μL) were separately mixed with 20 μL bacterial suspension and incubated at 37°C for 1 h. Samples were diluted 100-fold, spread onto plates, and incubated overnight, and colonies on plates were counted. The bacterial survival rate was calculated using the following formula:

Bacterial survival rate = (number of colonies with normal serum/number of colonies with inactivated serum) × 100%.

AB ATCC17978 served as the control strain.

### Cell adhesion test

An overnight bacterial culture was diluted to OD_600_ = 0.5, and 100 μL was placed in a 24-well cell culture plate containing A549 lung epithelial cells at 80% density and incubated at 37°C for 5 h. Non-adhered cells were washed with sterile PBS, incubated with 1% Triton X-100 for 5 min, and diluted 10-fold, and the number of viable bacteria were counted. AB ATCC17978 served as the control strain.

### G. mellonella *infection experiment*

An overnight bacterial culture was diluted to a cell density of 1 × 10^6^ CFU/mL, and *G. mellonella* individuals weighing ~250 mg were randomly divided into groups with 15 individuals in each group. Each individual was injected with 20uL of a 1 × 10^6^ CFU/mL bacterial suspension, incubated at 37°C, and assessed once every 8 h for 7 days. The acupuncture method was used to judge survival status, and death was defined as no response in the test. The Kaplan-Meier estimator method was used to plot a survival curve for *G. mellonella*. PBS served as the negative control, and AB ATCC17978 was served as the control strain.

### Mouse infection experiment

An overnight bacterial culture was diluted to a cell density of 1 × 10^8^ CFU/mL and used to infect 6-week-old BAMH/C immunocompromised mice by supernatant atomization [32]. Mice were then observed for 7 days after infection, and a survival curve was plotted using the Kaplan-Meier estimator method. The control strain was AB ATCC17978.

### *RT-qPCR measurement of* wzb, wzc, *and* wzi *expression*

A 3 mL overnight bacterial culture was centrifuged, and bacterial RNA was extracted using a PureLink RNA Mini Kit (Invitrogen) following the manufacturer’s instructions. RNA was reverse-transcribed using a reverse transcription kit (TaKaRa) to obtain cDNA and RT-PCR was performed using an RT-PCR kit (Bio-Rad). Expression levels of *wzb,wzc* and *wzi* were determined based on the results of RT-qPCR. 16sRNA was used as an internal reference . The control strain was AB ATCC17978. The primers are listed in Supplementary Table S2.

### Statistical analysis

For statistical analysis, values are presented as means±SD (standard deviation). Data are given as mean±standard deviations from three independent experiments. Rank-sum test was performed for pair-wise comparisons of groups. A two-tailed *P* < 0.05 was considered significant. The Kaplan-Meier method was used to estimate the survival distribution function.

## Results

### *Construction of* wza *knockout and complementation strains*

**Figure 1.**
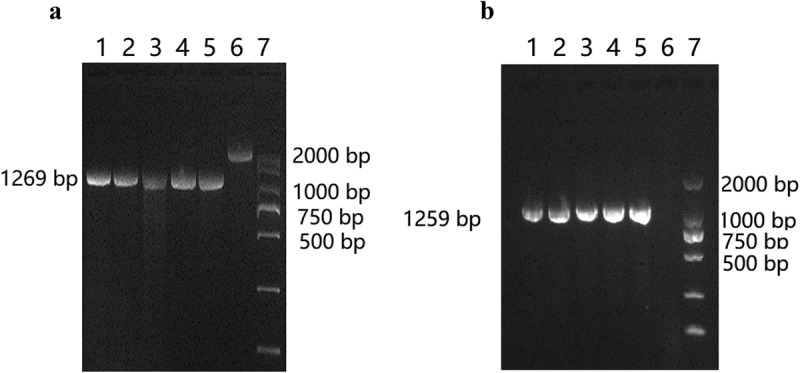
Detection of *Wza* knockout and complementation strains. **(a) Detection of *Wza* knockout strains**. Lane 1–4: the amplified fragment was 1269 bp, comprising the 622 bp upstream fragment and the 647 bp downstream fragment were amplified from the *wza* knockout strain. Lane 5: upstream and downstream regions of the *wza* gene were amplified from pLP-*wza* recombinant plasmid control. Lane 6: upstream and downstream regions of the *wza* gene were amplified from the WT strain, the size of the fragment was 2373 bp. Lane 7: DL2000 DNA Maker. **(b) Detection of *Wza* complementation strains**. Lane 1–4: the amplified 1259 bp fragment consists of the 1104 bp *wza* gene and 155 bp from the w*za* complementation strains. Lane 5: the amplified 1259 bp fragment consists of the 1104 bp *wza* gene and 155 bp from pBAD33-TC^R^ vector control. Lane 6: the amplified fragment of *wza* gene from the w*za* complementation strains. Lane 7: DL 2000 DNA Marker

As shown in Figure 1(a) (lane 6), upstream and downstream regions of the *wza* gene were amplified from the WT strain. The entire 2373 bp fragment comprises 622 bp upstream of the *wza* gene, the 1104 bp *wza* gene itself, and 647 bp downstream of the *wza* gene. The *wza* knockout strain was successfully engineered, as shown in lanes 1–4; the amplified fragment is 1269 bp, comprising the 622 bp upstream fragment and the 647 bp downstream fragment. As shown in Figure 1(b) (lanes 1–4), the complementation strain was also successfully obtained; the amplified 1259 bp fragment consists of the 1104 bp *wza* gene and 155 bp from the pBAD33-TC^R^ vector. The DNA sequencing results further confirmed that both *wza* knockout and complementation strains were successfully constructed.

### Growth kinetics

**Figure 2.**
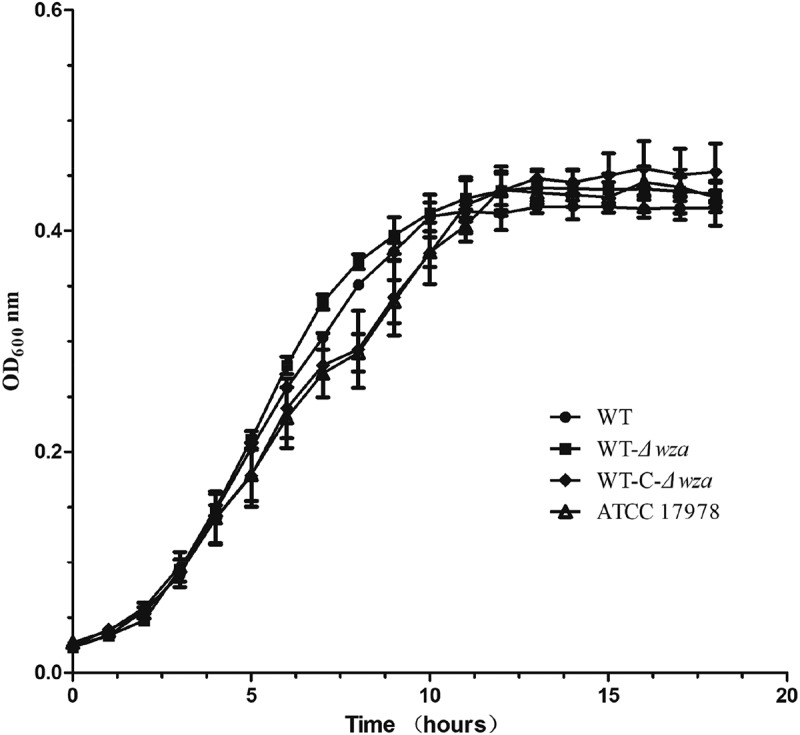
Growth (OD600) of A. baumannii strains cultured at 37°C in MH broth. WT is wild type strain, WT-*Δwza* is wza knockout strain, WT-C-*Δwza* is *wza* replenishment strain, and ATCC17,978 is control strain. Error bars indicate the s.d. for the three biological replicates examined for each strain.

To compare the in vitro growth rates in different AB strains, growth kinetics were determined for each strain at 37°C in MH broth (Figure 2). In general, the growth rates of the *wza* knockout strain and *wza* complementation strain were similar to those of the wild type strains.

### Bacterial morphology and biofilm formation

**Figure 3.**
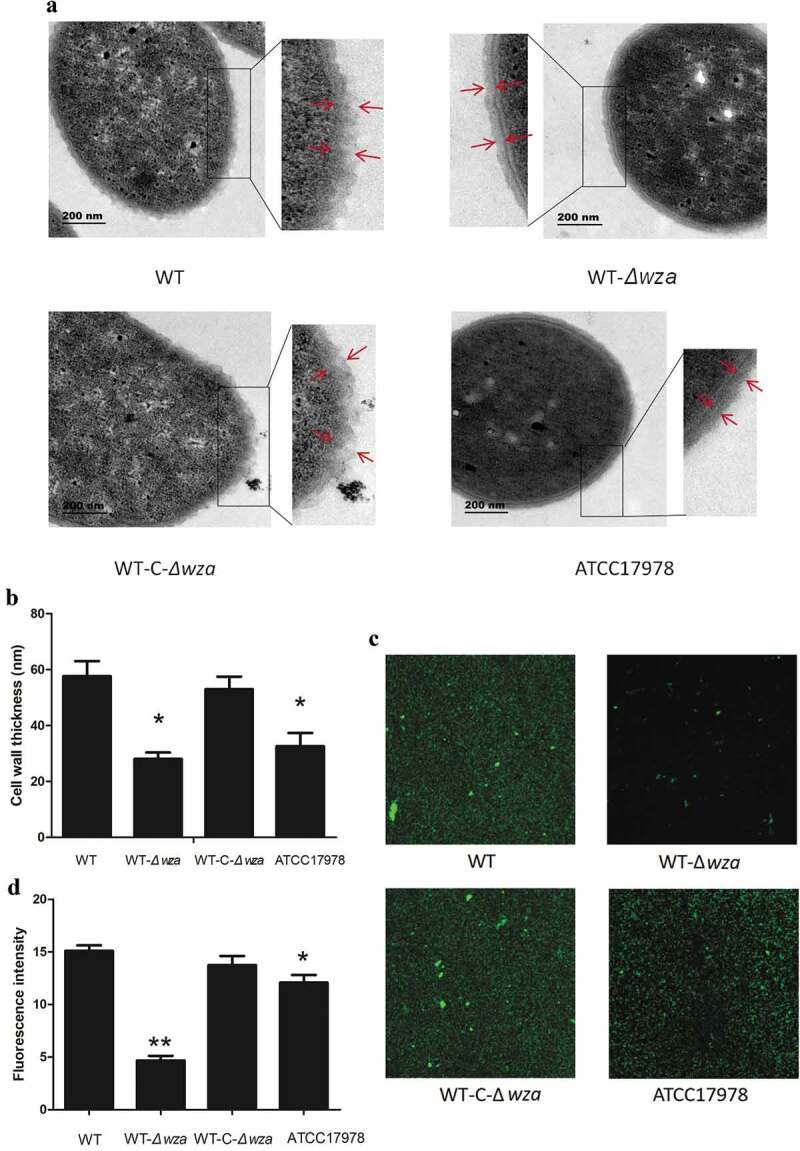
Bacterial morphology and biofilm formation. **(a) Observation of bacterial capsules by transmission electron microscopy (9800X). The red tip shows the cell wall of bacteria. (b) Statistical analysis of the thickness of bacterial cell wall. (c) Laser scanning Confocal Microscopy to observe biofilm formation**. Green fluorescence shows the biofilm of bacteria. The intensity of green fluorescence reflects the thickness of the biofilm. **(d) Statistical analysis of the intensity of green fluorescence**. WT is wild type strain, WT-*Δwza* is *wza* knockout strain, WT-C-*Δwza* is *wza* replenishment strain, and ATCC17978 is control strain Error bars indicate the s.d. for the three biological replicates examined for each strain. Asterisks indicate statistically significantly different from SKLX024256 WT (*P < 0.05, **P < 0.01, one-way unpaired analysis of variance, n = 3).

TEM was employed to visualize the cell wall of *wza* knockout and complementation strains. As shown in Figure 3(a), the cell wall of the *wza* knockout strain was smoother than that of the WT strain. Additionally, the thickness of the cell wall of the *wza* knockout strain was 32.26 nm, significantly lower than that of the WT strain (61.48 nm; *p* = 0.003; Figure 3(b)). We found the *wza* gene knockout to be deficient in bacterial capsule. Furthermore, the thickness of the cell wall of the *wza* complementation strain was returned to that of the WT strain, and the cell surface recovered its rough appearance. Moreover, the cell wall thickness of the WT strain was thicker than that of the AB ATCC17178 control strain (37 nm; *p* = 0.011). The confocal fluorescence microscopy results also showed that the fluorescence intensity of the *wza* knockout strain was weaker than that of the WT strain (*p* < 0.01; Figure 3(d)), while the fluorescence intensity of the *wza* complementation strain was restored to the level of the WT strain. Biofilm growth was also reduced in the AB ATCC17978 control strain compared with the WT strain. These results demonstrate that the *wza* gene affected the growth of bacterial biofilms.

### *Cell adhesion, anti-complement killing, and anti-oxidation* in vitro

**Figure 4.**
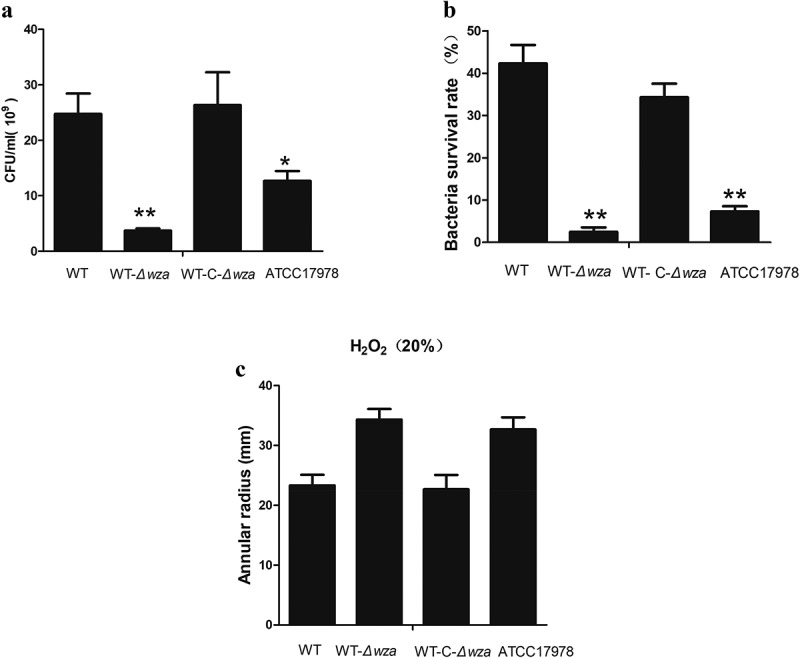
Cell adhesion, anti-complement killing, and anti-oxidation in vitro. **(a) The adhesion ability of the bacteria in vitro. (b) Anti-serum complement killing of bacteria in vitro. (c) anti-oxidation of bacteriain in vitro**. WT is wild type strain, WT-*Δwza* is *wza* knockout strain, WT-C-*Δwza* is *wza* replenishment strain, and ATCC17978 is control strain(*P < 0.05, **P < 0.01, one-way unpaired analysis of variance, n = 3).

The A549 non-small cell lung cancer cell line was used as an *in vitro* bacterial adhesion model, and the results showed that knockout of the *wza* gene resulted in significantly lower adhesion than observed with the WT strain (3.69 × 10^8^ vs. 2.47 × 10^9^; *p* = 0.005). Furthermore, cell adhesion of the *wza* complementation strain covered to the level of the WT strain. Similarly, the adhesion ability of the AB ATCC17178 strain was significantly lower than that of the WT strain (*p* < 0.01; Figure 4(a)). This indicates that the *wza* gene is involved in the bacterial adhesion process.

As shown in Figure 4(b), the survival rate of the WT strain was 42.3% at 90% normal serum concentration, while the survival rate of the *wza* knockout strain was only 2.5% under the same conditions (*p* = 0.001). These results indicate that the *wza* gene is important for resistance to complement-related killing. In another experiment, we used 20% H_2_O_2_ to test the tolerance of bacteria to oxidants. The result showed that the diameter of the inhibition zone of the WT strain was lower than that of the *wza* knockout strain (23.3 mm vs. 34.3 mm; *p* = 0.070), but the difference was not significant. This indicates that tolerance to 20% H_2_O_2_ was decreased following loss of the *wza* gene. The inhibition zone diameter of the complementation strain was 22.7 mm. Thus, the *wza* gene appears to be involved in the antioxidant process, at least *in vitro*. Additionally, the WT strain was more tolerant to 20% H_2_O_2_ than the AB ATCC17978 control strain (*p* = 0.108).

### *Bacterial infection of* G. mellonella *and mice*

**Figure 5.**
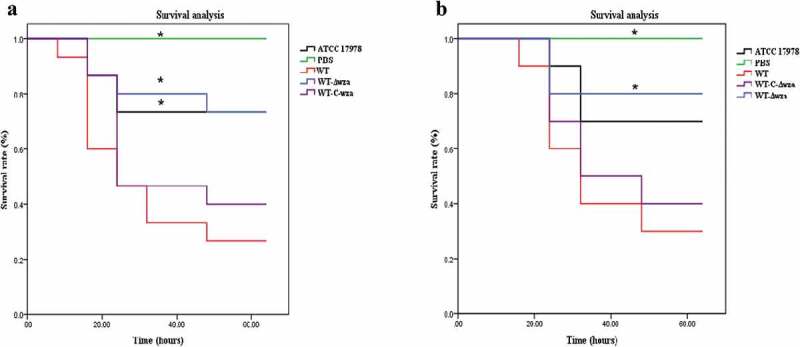
Bacterial infection of *G. mellonella* and mice. **(a) Kaplan-Meier curve analysis of the survival of Galleria mellonella after infection. (b) Kaplan-Meier curve analysis of the survival of mice after infection**. WT is wild type strain. WT-*Δwza* is wza knockout strain, WT-C-*Δwza* is wza replenishment strain, PBS is Phosphate Buffered Saline, and ATCC17978 is control strain(*P < 0.05, one-way unpaired analysis of variance, n = 3). Black lines is ATCC17978 infection, green line is WT infection, blue lines is WT-*Δwza* infection, red line is WT-C-*Δwza* infection.

As shown in Figure 5(a), *G. mellonella* died within 48 h following infection with AB. Mortality reached 73.3% following infection with the WT strain, and reached 53.3% within 24 h. By comparison, mortality following infection with the *wza* knockout strain was 26.7%, and was only 20% within 24 h. Kaplan-Meier survival curve analysis showed that the mortality of *G. mellonella* was significantly lower when infected with the *wza* knockout strain (*p* = 0.010). Furthermore, infection with the AB ATCC17978 control strain was significantly lower than with the WT strain (*p* = 0.031). Similar results were obtained for the mouse infection experiment (Figure 5(b)). The mortality of mice infected with the WT strain reached 70% and the mortality rate was 40% within 24 h. By contrast, the mortality of mice infected with the *wza* knockout strain was only 20%. Kaplan-Meier survival curve analysis showed that the mortality of mice infected with the *wza* knockout strain was significantly lower than that of mice infected with the WT strain (*p* = 0.033).

### *Expression of* wzb, wzc, *and* wzi *in* wza *knockout and complementation strains*

**Figure 6.**
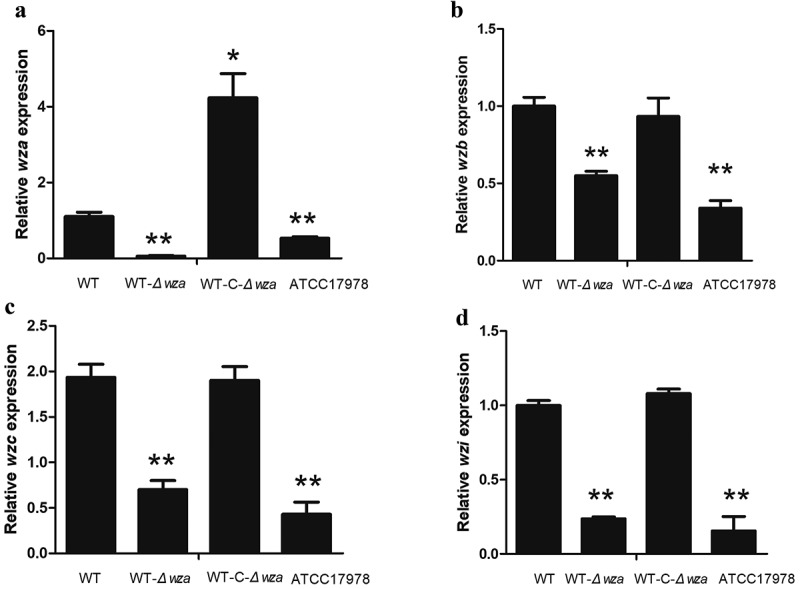
Expression of *wza, wzb, wzc, and wzi* in *wza* gene knockout and complementation strains. **(a) Detection of *wza* expression in strains by RT-PCR. (b) Detection of *wzb* expression in strains by RT-PCR. (c) Detection of *wzc* expression in strains by RT-PCR. (d) Detection of *wzi* expression in strains by RT-PCR**. WT is wild type strain, WT-*Δwza* is *wza* knockout strain, WT-C-*Δwza* is *wza* replenishment strain ((*P < 0.05, one-way unpaired analysis of variance, n = 3, **P < 0.01, one-way unpaired analysis of variance, n = 3).

Analysis of the expression levels of genes up- and downstream of *wza* showed that *wzb* (*p* = 0.003), *wzc* (*p* = 0.002), and *wzi* (*p* = 0.002) were significantly decreased in the *wza* knockout strain, and expression of all three genes was restored to WT levels in the *wza* complementation strain (Figure 6). These results indicate that the Wza protein affects the expression of *wzb, wzc*, and *wzi* in the Wzy transport pathway.

## Discussion

*Acinetobacter baumannii* is an important nosocomial pathogen worldwide that causes a variety of serious infections. Due to the extensive use of antibiotics, the detection rate of carbapenem-resistant, multi-drug-resistant, and even totally drug-resistant strains is increasing, and control of infections faces enormous challenges. Unlike other Gram-negative opportunistic pathogens such as *E. coli* and *K. pneumoniae*, AB has long been considered an opportunistic pathogen with low virulence, but reports of highly virulent strains are increasing, consistent with an increase in their prevalence. Thus, it is important to study the virulence factors and virulence mechanisms of AB to prevent and control possible epidemics. An in-depth study of the virulence of AB could result in new treatments for multi-drug-resistant AB infections.

In the present study, the MLST of the SKLX024256 strain was ST195, belonging to the CC92 clone complex. At present, ST191, ST195, and ST208 are the most prevalent strains of carbapenem-resistant AB in China [33]. Studies have shown that ST195 clinical isolates are positive for biofilm formation genes (such as those encoding Csu and Bap), and also carry virulence genes such as *pga* and *ompA*, which are rare in less virulent isolates. Additionally, biofilm formation, phospholipase C production, hemolytic activity, and toxin production are higher than in non-virulent strains. This indicates that clinically isolated ST195 strains possess high virulence [34].

Highly virulent AB strains have stronger biofilm formation, cell adhesion, and cell invasion abilities than non-virulent strains [35], and these abilities are closely related to the bacterial capsule. Capsular polysaccharide has many important functions in bacteria [1]: it plays an important role in the fight against phagocytosis by phagocytes in the host immune system [36, 2] it promotes adhesion between bacteria, and between bacteria and cells, thereby promoting biofilm formation and colonization in different living environments [37]; and [3] it protects bacteria against harmful substances such as host lysozyme and complement factors.

Studies have shown that Wza and OmpA are expressed strongly in a variety of highly toxic AB strains [38]. Both Wza and protein tyrosine kinase (PTK, Wzc) genes are essential for the polymerization and assembly of capsular polysaccharides, and when mutated using transposons, capsule integrity is compromised [18]. In the present study, we constructed a *wza* knockout strain, and found the strain to be capsule deficient with the thickness of its cell wall reduced by 50% compared with the WT strain, and biofilm formation was reduced. These results suggest that Wza affects the formation of bacterial capsules. Russo et al constructed the *wza* mutant and found that the *wza* mutants were capsule deficient [18], Skerniškytė J et al also constructed a *wza* mutant and found it was a CPS-negative phenotype [26]. These studies were consistent with the results of the cell wall we assessed by TEM. Previous bacterial studies also showed that expression of Wza is positively correlated with the expression of capsular polysaccharides, and deletion of this gene in *Lactobacillus johnsonii* completely prevents capsular polysaccharide formation [39]. Furthermore, a recombinant plasmid carrying the gene encoding wza completely restored extracellular polysaccharide biosynthesis [39]. Additionally, when the *wza* gene is mutated in *Bacillus amylolytica*, biofilm formation is reduced, and biofilm structure is incomplete [40].

In a previous study, normal mice died within 48 h of infection with the highly virulent AB5075 strain at a dose of 1.0 × 10^5^ CFU/mL, but survival was decreased to 33% when infected with a *wza* mutant strain [3]. In our study,we found the *wza* knockout strain had significantly lower cell adhesion (3.69 × 10^8^ vs. 2.47 × 10^9^; *p* = 0.005) and significantly lower survival compare to that of WT (2.5% VS 42.3%, *p* = 0.001), but the growth rate displayed no difference. These results were consistent with Russo et al 2010 results [18]. These results suggest that *wza* knockout may disrupt the capsule on the bacterial surface, which may weaken bacterial resistance to harsh external environments and cell adhesion ability, thereby weakening the ability of bacteria to survive and infect the host. Furthermore, our *in vivo* virulence tests showed that the mortality of *G. mellonella* and mice infected with the *wza* knockout strain was significantly lower than that of animals infected with the WT strain.

Wzy capsular polysaccharide synthesis-dependent pathway is crucial for capsular polysaccharide assembly [41]. Ribonucleotide precursors in the cytoplasm assemble four monosaccharide repeating units of the polymer through a series of enzymes including integral membrane proteins and peripheral membrane protein lipids (undecanoic acid diphosphate; und-PP receptor) [42]. The und-PP-linked repeating units are flipped through the inner membrane by the Wzx protein. Under the action of Wzy, polymerization on the periplasmic surface forms long-chain polymers, and high-level Wzy-dependent polymerization requires activation of the tetrameric Wzc protein [43]. The Wza protein undergoes autophosphorylation to facilitate assembly of the capsular polysaccharide [44]. The Wzb phosphatase anchors the catalytic domain on the surface of Wzc, dephosphorylates Wzc, and controls Wzc phosphorylation and dephosphorylation, thereby regulating the degree of polymerization and yield of capsular polysaccharide [45]. Polymers exported to the surface require the outer membrane Wza eight polymer complex, and Wza and Wzc proteins interact to form a complex that spans the periplasm [46–48]. Thus, the activities of Wzc and Wzb play an important role in the synthesis of capsules. Reports indicate that co-expression of these two proteins is essential for capsule assembly in *E. coli* K30 [49,50].

Additionally, Wzc regulates the export of capsular polysaccharide by controlling the opening and closing of the Wza protein [51,52]. Studies have shown that the Wzi protein is a key factor in the initial extracellular anchoring of capsular polysaccharide [53]. This creates a template for carbohydrate transfer to hydrophobic adventitia to form capsules. Wzi acts as an agglutinin on the cell surface and combines with capsular polysaccharides immediately after transport through Wza. Capsular polysaccharides can be synthesized and transported to extracellular sites, but cannot form capsules with the functional Wzi outer membrane protein [54,55].

Based on the bacterial toxicity test results of our study, Russo et al. 2010 [18] and Skerniskyte et al. 2019 [26], we found that after *wza* knockout, bacteria couldn’t form capsule layer, which was easily killed by the immune system of the host, and what effect does *wza* have on the wzy pathway? whether the absence of Wza affects the expression of other molecules involved in the synthesis of Wzy capsular polysaccharides has not been reported. We found *wza* knockout led to deceases in the expression of wzb, wzc, and wzi, suggesting that knockout of *wza* gene, the bacterial capsular polysaccharide can not be transported to the outer membrane, in order to avoid the accumulation of excessive capsular polysaccharide in the cells, the expression of the capsular export genes are down-regulated. This indicates that Wza not only controls the output channel of capsular polysaccharides, but also indirectly affects the expression of these three proteins that are crucial for the Wzy capsule formation pathway, thereby affecting the synthesis, transport, and extracellular fixation of capsular polysaccharides. Complementation of the *wza* gene restored expression of these three genes to WT strain levels

## Conclusions

Herein, we constructed *wza* knockout and complementation strains using clinical isolates of AB SKLX024256. Our results showed that after *wza* knockout, the capsule is deficient and the thickness of its cell wall is reduced, biofilm formation ability is weakened, adhesion to A549 cells is diminished, and resistance to serum complement killing and oxidants is compromised. The mortality of *G. mellonella* and mice was also significantly decreased following infection with the *wza* knockout strain, compared with the WT strain. Therefore, Wza is a conserved capsular polysaccharide transport protein affecting the formation of the bacterial capsules. It is important for the virulence of AB, and provides a potential target for new antibacterial therapies.

## Supplementary Data

The supplementary data for this article can be accessed here.
