# Supplementary material for: Wza gene knockout decreases Acinetobacter baumannii virulence and affects Wzy-dependent capsular polysaccharide synthesis
Source: Virulence. 2019 Dec 27;11(1):1–13. doi: 10.1080/21505594.2019.1700659 (PMC6961727; doi:10.1080/21505594.2019.1700659)
Supplement: Supplemental Material [file kvir-11-01-1700659-s001.docx]

**Table 1S. Description of primers used for PCR and DNA sequencing**

| Primer name | Sequence |
| --- | --- |
| *wza*-MF1 | GGAATCTAGACCTTGAGTCGGCACATACAACGCTTAAAATAACGC |
| *wza*-MR1 | TCGCGTGTGAAGTATTGCCAGCAGATTATCCCGTTCTCAAATGCTT |
| *wza*-MF2 | AAGCATTTGAGAACGGGATAATCTGCTGGCAATACTTCACACGCGA |
| *wza*-MR2 | ACAGCTAGCGACGATATGTCTTAACTTGGTCCACAGGTGTAGGG |
| pLP-UF | GACACAGTTGTAACTGGTCCA |
| pLP-UR | CAGGAACACTTAACGGCTGAC |
| *wza*-TF | CTGTCCTCAACCTGAACTAAAGCAT |
| *wza*-TR | AGAGCTTGACCAACTGTCTCGC |
| *wza*-RF | TGGGCTAGCGAATTCGAGCTAGGAGGAATTCACCTTATTGTCCCAAACG |
| *wza*-RR | TGCCTGCAGGTCGACTCTAGGTGAAGTATTGCCAG |
| pBAD33-TC^R^-ZF | CCATAAGATTAGCGGATCCTACCT |
| pBAD33-TC^R^-ZR | CTTCTCTCATCCGCCAAAACAG |

**Table 2S. Description of primers used for RT-PCR**

| Primer name | Sequence |
| --- | --- |
| *wza*-RT-F | GCAGCGCTTCTCTGTTCAAG |
| *wza*-RT-R | TCACCTGTTGTAGTGACGCC |
| *wzb*-RT-F | ATTGGGCATCAAGCCGATGA |
| *wzb*-RT-R | AGCTCGGCATTGAGCTTTCT |
| *wzc*-RT-F | CACCCAGCAATGCGTGAAAT |
| *wzc*-RT-R | GGTTCAACTGGCTCAACTGC |
| *wzi*-RT-F | TATAAAGCGGTCCCGGATGC |
| *wzi*-RT-R | GTCCATCTCCTCCCCACTGA |
| 16s-RT-F | GGAGGAAGGTGGGGATGACG |
| 16s-RT-R | ATGGTGTGACGGGCGGTGTG |
